# Effect of Friction Stir Welding Techniques and Parameters on Polymers Joint Efficiency—A Critical Review

**DOI:** 10.3390/polym13132056

**Published:** 2021-06-23

**Authors:** Miguel A. R. Pereira, Ana M. Amaro, Paulo N. B. Reis, Altino Loureiro

**Affiliations:** Department of Mechanical Engineering, CEMMPRE, University of Coimbra, 3030-788 Coimbra, Portugal; miguelreispereira@outlook.pt (M.A.R.P.); paulo.reis@dem.uc.pt (P.N.B.R.); altino.loureiro@dem.uc.pt (A.L.)

**Keywords:** friction stir welding (FSW), joint efficiency, stationary shoulder, external heating, double-side passage, polymeric materials

## Abstract

The objective of current work is to analyse the influence of different welding techniques and welding parameters on the morphology and mechanical strength of friction stir welds (FSW) in polymers, based on data collected in the literature. In the current work, only articles that provide data on the joint efficiency, or sufficient information to estimate it are considered. The process using conventional tool is presented and compared with new procedures developed for FSW of polymers, such as those using tools with heated stationary shoulder, preheating of the polymer or double-side passage of the tool. The influence of tool rotational speed (w), welding speed (v), tilt angle and geometry of the pin are discussed. This work focuses on the polymers most studied in the literature, polyethylene (PE) and polypropylene (PP). The use of external heating and tools with stationary shoulder proved to be of great importance in improving the surface finish, reducing defects, and increasing the mechanical strength of the welds. The increase in the *w/v* ratio increased the joint efficiency, especially when using conventional tools on PE. A trend was obtained for conventional FSW, but it was difficult to establish mathematical relationships, because of the variability of welding conditions.

## 1. Introduction

The importance of polymers has grown significantly in the industry due to the increase in the number of applications of these materials in daily life [1]. Low density, good moldability, excellent resistance to corrosion and low production costs are some of the characteristics of polymeric materials that have led to the substitution of many materials by polymers in many sectors of industry, especially in the automobile, naval and aerospace sectors [2]. There are countless cases in which it is not possible to manufacture the item in one single piece. Factors such as size, geometry, the coexistence of different materials or even due to the product’s own mode of operation require the division of the product into more than one component and, subsequently, the union of these to obtain the final product [3]. In the case of polymeric materials, the main joining methods are divided into mechanical fastening, adhesive bonding, and welding. Mechanical fastening causes stress concentration and is not watertight, while adhesive bonding is difficult to do, due to the reduced surface tension of the polymers, besides being sensitive to the environment [4,5]. Welding currently involves the melting of a portion of material that, after cooling and solidifying, gives rise to the welded joint [1]. Welding processes can be divided into three different categories, depending on the melting process associated, which are thermal welding, friction welding and electromagnetic welding [1,6].

Many welding technologies have been tested in welding of polymers in recent years, of which the most notable are: laser welding (LW) [7,8], ultrasonic welding (USW) [9], induction welding (IW) [10] and friction stir spot welding (FSSW) [11]. LW depends a lot on the optical properties of the polymers, USW is limited to reduced thicknesses of the joints and IW requires long process times. FSSW and friction stir refill spot welding (FSpW) are variations of FSW where there is no linear movement of the tool, which only allows spot welding, resulting in the stress concentration and does not guarantee a watertight weld. In addition, all these processes produce welds with relatively low joint efficiency. Actually, Pereira et al. [12] refer to a maximum joint efficiency of 50% for the Nd: YAG Laser welding of double-bead lap joints in PA, while Oliveira et al. [13] report a joint lap-shear strength of about 9.5 MPa for FSpW of lap joints in polymethyl methacrylate (PMMA) and Zhu et al. [14] a joint lap-shear strength that does not exceed 2.5 MPa in thermal bonding of PMMA.

**Figure 1:**
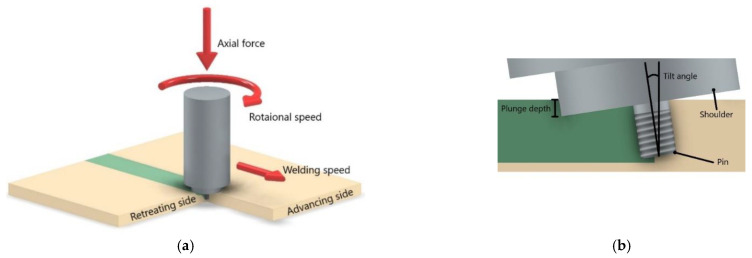
Schematic illustration of the FSW process in (**a**) isometric view and (**b**) section view.

Friction Stir Welding (FSW) is one of the welding methods within the category of friction welding, as it uses the friction between the base material and the tool to generate the heat necessary to soften the material of the joint [15]. The FSW process was developed, demonstrated and patented by “The Welding Institute (TWI)” in England for the first time by Thomas et al. [16,17]. The principle of the conventional process is illustrated in Figure 1, as well as its main variables. These main variables are the rotational speed, welding speed (or traverse speed), tilt angle (or attack angle), axial force and plunge depth (or penetration depth). The conventional tool consists of a shoulder and a pin that rotate together, generating heat that is responsible for heating and softening the material to be welded. The pin is also responsible for mixing the softened material in the weld seam, while the shoulder prevents the projection of material out of the welding zone [2]. FSW does not require a filler material, protective atmosphere or joint preparation [17] and does not generate toxic fumes nor UV radiation, so it is considered environmentally friendly [18]. Since the main source of heat is the friction between the base material and the tool, beyond the energy of plastic deformation of the material [19], FSW is an economic and energy efficient process [20]. Butt and lap joint configurations are the most common in FSW, although other configurations can be used [2]. The FSW for lap joint configuration is also known as Friction Stir Lap Welding (FSLW) [21].

This welding method was developed to overcome the difficulties of traditional welding techniques when applied to lightweight alloys and was initially used to weld aluminium alloys. After proving its potential, FSW was also applied to other metals and their alloys, such as magnesium, copper, titanium and steel [2,22], and it was first demonstrated on polymers in 1997 [1].

For metals, FSW is defined as a solid-state welding process since, typically, the melting temperature is not reached during the FSW [23]. In the welding of polymers, Inaniwa et al. [24] mentioned that the melting temperature is never reached during FSW of 5 mm thick plates of high density polyethylene (HDPE), polyamide 6 (PA6) and polyvinyl chloride (PVC) in butt joint configuration. However, Strand [15] argued that the FSW of polymers is not a process that occurs exclusively in the solid state. Polymeric materials have a temperature range in which the change of phase occurs, because they are composed by molecular chains with different lengths and molecular weights and therefore, with different melting temperatures. According to this researcher, during the FSW of polymers the shortest molecular chains reach the melting temperature, while the largest do not. For this reason, FSW in polymeric materials was redefined as a welding process in which there are portions of solid material suspended in a melted polymeric matrix. During the FSW of 15 mm thick HDPE plates on butt joint configuration, the temperatures measured by Rezgui et al. [23] were between 128 °C and 250 °C, which, according to the author, was above the melting temperature of this polymer and that also proved that the welding process occurred in the liquid state. Similar conclusions were presented by Bozkurt [25] after measuring temperatures between 120 and 165 °C while welding 4 mm thick HDPE plates in the butt joint configuration. Eslami et al. [26], during the FSW of 3 mm thick high molecular weight polyethylene (HMW-PE) plates in butt joint configuration, also measured temperatures above the welding temperature of the polymer which according to him, reinforces Stand’s theory [15].

FSW and other welding processes cannot be applied to all types of polymers because thermosetting polymers show irreversible transformations when exposed to high temperatures, due to the change in their molecular structure, even below the melting temperature. On the other hand, thermoplastic polymers soften when exposed to a temperature increase, without a degradation of their molecular structures. They are also known as recyclable polymers, due to the possibility of being moulded more than once [2,27]. Therefore, FSW is only applicable to thermoplastics [28]. This technique has successfully joined: acrylonitrile butadiene styrene (ABS) [29,30,31,32], polyamide 6 and 66 (PA6 and PA66) [33,34,35], polycarbonate (PC) [36,37], polyethylene (PE) [25,38,39], polylactic acid (PLA) [40], polymethylmethacrylate (PMMA) [41,42,43], polypropylene (PP) [44,45,46] and polyvinyl chloride (PVC) [24].

New techniques have been developed in recent years to resolve the problems identified concerning the conventional FSW of polymers. For example, the root defect corresponds to an area at the bottom of the joint that is not welded, because the heating of that zone was inadequate [15]. Therefore, the lack of welding at the root of the joint results in a decrease in the tensile and flexural strengths of the weld [47]. To solve this problem of the root defect, which frequently occurs in conventional welding, Arici and Sinmaz [47] performed welding on both sides of the polymers to be joined (double-side passage), thus completely eliminating this defect.

Another innovation related to the FSW of polymeric materials was the introduction of stationary shoulders. According to Strand [15], a tool with a rotating shoulder is not able to retain the polymeric material inside the weld seam as happens in the FSW of metals. Stationary shoulder tools have been developed to eliminate this phenomenon. The solution found consists of the application of bearings between the shoulder and pin, which allows the pin to be the only rotating element and the shoulder to slide smoothly on the weld surface. With the introduction of non-rotating shoulders, it was possible to reduce the amount of material pushed out of the weld volume and to improve the surface finish of the weld significantly. The use of stationary shoulder tools is also beneficial to the FSW process for polymeric materials, specially to solve the peeling effect and the rough surface finish of the conventional process [2]. When the stationary shoulder is long and rectangular, it is called a shoe [15].

Because FSW operates at lower temperatures than conventional welding methods, heat conduction plays a very important role in the performance of this process. Since polymers have low thermal conductivity, mainly due to their molecular structure, heat conduction in polymeric materials is less efficient, which makes it difficult to achieve strong FSW joints [19]. To overcome this difficulty Aydin [3] preheated the polymer before welding which allowed them to remove any cavities and improve the joint efficiency.

**Figure 2:**
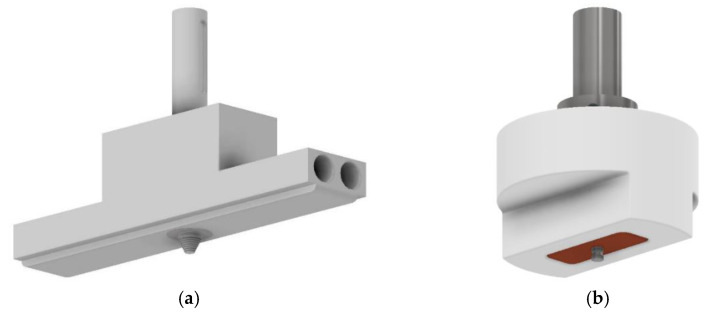
(**a**) FSW tool with stationary shoulder, with external heating (adapted from [31]) and (**b**) FSW tool with stationary shoulder, without external heating (adapted from [26]).

Depending on the case, preheating the material can be a difficult task, especially for large plates or long joints. Nelson et al. [48] reported that the use of a conventional tool provides welds in polymers with inadequate morphology and mechanical resistance and patented a tool with a stationary shoulder and heated by electrical resistances. Mendes et al. [31] developed a long stationary shoulder tool with a conical threaded pin in the centre of it, which can be used with or without electrical heating, and mounted on a robot [49]. Because the pin is in the centre of the shoe, the cooling stage may be controlled, as with other versions of hot shoe tools, but with better preheating of the material. The geometry of this tool is shown in Figure 2a. The shoulder is made of aluminium, to ensure good thermal conductivity, and the cylindrical holes serve to mount electrical resistances.

Recently, Eslami et al. [26] developed a very simple FSW tool with a stationary shoulder made of Teflon, without external heating, but where part of the heat is generated by the friction between a copper sleeve and the pin, and not just between the pin and the polymer. A scheme of the tool is shown in Figure 2b.

FSW of polymeric materials continues to be studied and is responsible for a very significant number of published papers, including some reviews of relevant interest [50,51,52,53,54]. However, most of these papers describe the technology of each process/variant and/or its direct effects on welds, but they do not compare the effects of different technologies, mainly in terms of the mechanical strength of the joints. In this context, and due to the great scientific and industrial interest, this paper is intended to be a first attempt to fill this gap. Therefore, the aim of this study is to analyse, compare and summarize the effects of conventional and alternative FSW techniques and tools, as well as the main process parameters, on the quality of welds in thermoplastic polymers based on the literature published. For this purpose, a graphic analysis was developed to summarise the information, in order to show the main trends, although the task is difficult due to the great variability of the experimental conditions described in the literature. The focus was mainly directed at PE and PP because they are the polymers most welded by FSW. At the same time, a chronological review of the use of this process in the welding of polymers is carried out. The bibliographic search that supports the current study focused mainly on papers that show the efficiency of the joints, or that provide enough information for their estimation. Although more than a hundred articles were consulted, only a quarter of the articles provide the weld joint efficiency or sufficient information for its estimation, which is insufficient for statistical treatment. Data were divided into the following categories taking in consideration the tool or procedure used: conventional tool, conventional heated tool, tool with stationary shoulder, tool with heated stationary shoulder, double-side pass, and preheated polymer. For PP there is only information available on some of these categories.

## 2. Graphical Presentation of Data

As mentioned above, the quality of FSW welds, and consequently their mechanical strength, depends on many parameters, so the analysis of their influence is a complex task. Therefore, it was decided to use in the current study the ratio between the speed of rotation (w) and feed (v) of the tool, because they are two of the main parameters of the FSW process. Furthermore, the increase in the rotational speed and the decrease in the welding speed increase the frictional heat input in the weld, which, as will be seen later, has a crucial influence on the quality of the welds.

On the other hand, joint efficiency is used in the current analysis to evaluate the strength of the welds, because it is a parameter available in many papers, or at least the strength of the welded joints is given, which allows the calculation of the joint efficiency. The joint efficiency is the ratio between the mechanical strength of the weld and the strength of the base polymer. Flexural strength, elongation at break, crystallinity and hardness were also evaluated in some studies, but with much less frequency. Although these results were not used to establish a direct comparison between studies, their influence was also considered in the current article.

**Figure 3:**
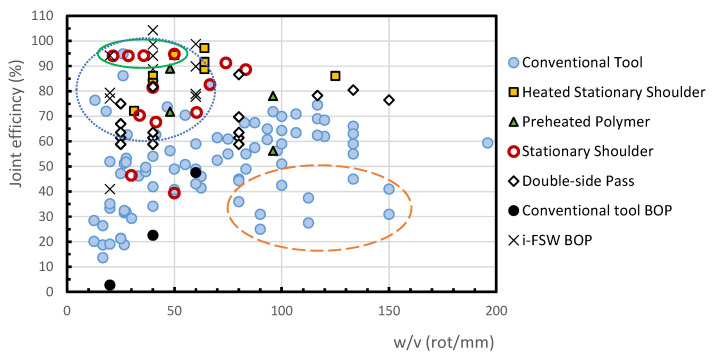
Effect of the *w/v* ratio on the joint efficiency of FSWs in PE.

**Figure 4:**
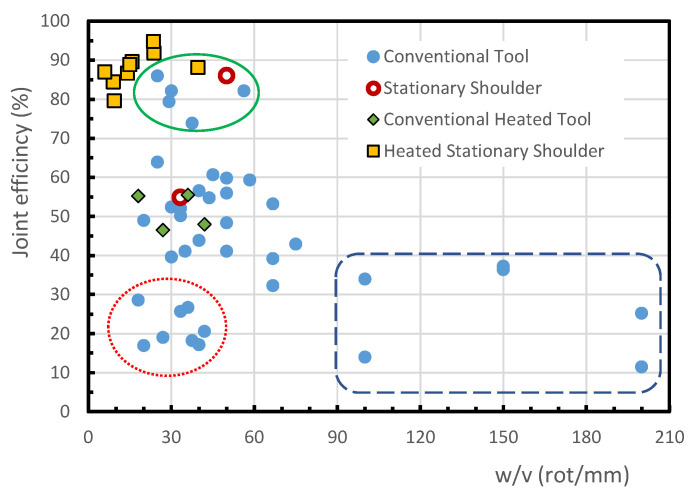
Effect of the *w/v* ratio on the joint efficiency of FSWs in PP.

The effect of the tool rotational speed to the welding speed ratio (*w/v*) on the joint efficiency of the welds of PE and PP is shown in Figure 3 and Figure 4, respectively. In the current study, medium density polyethylene (MDPE), high-density polyethylene (HDPE), high molecular weight polyethylene (HMW-PE) and ultra-high molecular weight polyethylene (UHMW-PE) were included in a generic category called PE.

Figure 3 and Figure 4 include the results of joint efficiency of welds produced by FSW process using conventional tools with and without external heating, welds with stationary shoulder tool, with and without external heating, welds with preheating of the base polymer and welds with double welding passage. The conventional process is one that is carried out with a conventional tool, that is a tool composed by a shoulder and a pin rotating together, as mentioned above.

As the (*w/v*) ratio does not consider the heat input by external sources, such as when using tool with heated stationary shoulder or previously heated polymer, these figures also allow comparisons between FSW with conventional tool and the innovations brought to the welding process. These aspects will be analysed in detail in the following sections.

## 3. Data analysis

### 3.1. FSW With Conventional Tool

#### 3.1.1. Welds in Polyethylene

In order to understand the effect of the technological innovations on the FSW of polymeric materials, it is important to firstly understand the performance of the conventional process. For the welding of PE, most of the data related to the conventional FSW process, illustrated by filled dots in Figure 3, show that by increasing the *w/v* ratio the joint efficiency tends to rise. A maximum value of joint efficiency of about 75% is reached for a *w/v* ratio of about 120 rot/mm (rotations per millimetre). Exception to this trend are the points with high joint efficiency, which are inside the dotted circle, that belong to a study carried out by Bozkurt [25], and the points with low efficiency, located within a dashed oval, of an investigation done by Saeedy and Givi [55]. These results are also analysed below.

The application of conventional FSW for 6 mm thick MDPE in butt joint configuration was investigated by Saeedy and Givi [56]. They analysed the influence of rotational speed and tool tilt angle on the mechanical properties of the joint. These researchers observed that the rotational speed had a greater impact on elongation and the tilt angle on the tensile strength. They observed that the tensile strength and elongation were higher with a tilt angle of 1°, a rotational speed of 1600 rpm and a welding speed of 15 mm/min, which means an optimum value for the *w/v* ratio of about 133.3 rot/mm. A maximum joint efficiency of 70% was achieved in these conditions. According to these researchers, it was not possible to achieve better results due to the occurrence of root defects and due to the insufficient heat generated, which also contributed to the formation of heterogeneous structures.

FSW on MDPE of 8 mm thick plates in butt joint configuration was performed to test the influence of tilt angle, rotational speed and welding speed, by Saeedy and Givi [55]. The optimum parameters found were a tilt angle of 1°, a rotational speed of 1400 rpm and a welding speed of 12 mm/min, which means a *w/v* ratio of about 116.7 rot/mm and a maximum joint efficiency of 74.7%. Lower rotational speeds were not sufficient to soften the polymer and higher rotational speeds led to flash defects, according to the authors. Higher welding speeds did not allow enough time to heat the polymer, and, in extreme cases, those speeds led to the milling of the material instead of welding it. A tilt angle of 2° led to the formation of tunnel defects, which reduced the weld’s strength. In Figure 3 the points below the tendency zone, which are inside a dashed oval, are related to this study and correspond to sets done with rotational speeds of 1800 rpm with a tilt angle of 1°, and rotational speeds of 1600 and 1800 rpm with tilt angles of 2°. The authors observed that in comparison with the base material there was a reduction in the crystallinity of the polymer in the welded region. According to them, lower welding speeds, and consequently higher heat inputs and longer cooling time led to the increase in the crystalline content and joint efficiency.

The application of FSW to several polymers was investigated by Inaniwa et al. [24]. Among them, 5 mm thick HDPE plates were welded by conventional FSW. In this case, a 0.1 mm gap was used between the shoulder and the plate surfaces, which means a negative plunge depth and a reduction in the compression on the softened material. In these conditions, no major defects were visible in the joint and a maximum joint efficiency of about 70% was obtained at a welding speed of 15 mm/min and a rotational speed of 1240 rpm, which corresponds to a *w/v* ratio of about 82.7 rot/mm.

The influence of the cooling stage after the conventional FSW process was investigated by Nateghi and Hosseinzadeh [57]. In this experiment, they butt welded 5 mm thick HDPE plates. The cooling was done with the application of CO_2_ at the pressure of 2 bar. They concluded that the rotational speed must be higher than 1000 rpm to assure a good mixing of the material, and less than 2200 rpm to avoid excessive temperature and, consequently, the degradation and burning of the polymer. The formation of wormhole defects in the retreating side (RS) was also observed. According to the authors, this defect occurred due to insufficient heat generation and consequent deficient mixing of the material, which is related to low rotational speeds. They also observed that for welding speeds below 40 mm/min, material degradation occurred, due to the excessive temperature, and for welding speeds above 80 mm/min, the low operating temperature coupled to the deficient mixing of the material resulted in a reduction of the weld’s strength. The welding speed of 40 mm/min and the rotational speed of 2200 rpm, which corresponds to a *w/v* ratio of 55 rot/mm were responsible for the maximum joint efficiency for the new and conventional processes. By applying CO_2_ at 2 bar, the maximum joint efficiency rose from 51% to 70%. The authors argue that by increasing the cooling rate, the thermal residual stress was reduced, which also led to a decrease in the angular distortion of the welds. These results suggest that the cooling rate influences the quality of the welds, although this parameter is not frequently considered.

A joint efficiency of just 44% for a *w/v* ratio of 80 rot/mm was reached by Mishra et al. [2] for butt welds of 6 mm thick HDPE plates, because a tool rotational speed of only 800 rpm was used. They also reported that the rotation of the shoulder led to the peeling of the surface and consequently, tiny voids were formed on the surface. According to them, FSW of polymers should be performed with stationary shoulders, to avoid this phenomenon.

The optimization of the conventional FSW of 4 mm thick HDPE plates, in butt joint configuration, by testing different tilt angles, welding speeds and rotational speeds, was done by Bozkurt [25]. A maximum joint efficiency of 94.9% was achieved with 3000 rpm of tool rotational speed, 115 mm/min of welding speed (*w/v* = 26 rot/mm) and a tilt angle of 3°. The points inside the dotted circle related to FSW with conventional tool in Figure 3 with joint efficiencies above 70% and a *w/v* ratio below 60 rot/mm are from this study. These points result from experimental sets performed with rotational speeds between 1300 rpm and 3000 rpm, welding speeds between 45 mm/min and 115 mm/min and tilt angles of 1°, 2° and 3°. The author stated that the root defect was prevented due to an improvement in the heat generated. The removal of this defect may justify the better results in comparison with other works. No information about the axial force, penetration depth or pin geometry used is available in this study. The correct choice of these parameters could also be one of the reasons for this researcher’s success.

No article was found with a comparative study of the influence of the pin geometry on the strength of welds in PE, however, it was found that welds with high joint efficiency were obtained with threaded pins [3,58,59] or grooved pins [26].

The strength of the welds is mainly controlled by the existence of defects, either lack of fusion at the root of the welds or cavities on the retreating side. It can therefore be concluded from the studies presented that the increase in the *w/v* ratio is beneficial for conventional FSW, because it increases the heat input and the plasticization of polymers, promoting the flow of material around the tool, thus reducing the root and internal defects in the welds. A research of one of the current authors [31] for welds in ABS plates confirms this trend. The use of a high *w/v* ratio does not guarantee that welds with a high efficiency will be produced, because the rotation of the shoulder causes the superficial degradation of the weld and excessive tool rotation speeds can burn the polymer. Figure 3 does not consider the influence of important parameters, such as tilt angle, axial force, tool plunge depth or tool pin geometry, that also affect the joint’s quality and strength, and that are the main cause of the dispersion of results shown in figure. The inclusion of these parameters was not possible, because it would make the figure more confusing.

#### 3.1.2. Welds in Polypropylene

**Figure 5:**
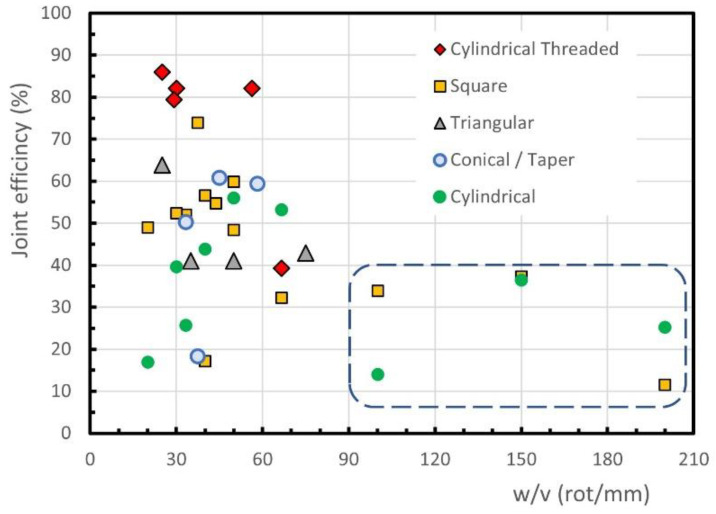
Effect of pin geometry on the joint efficiency for conventional FSW of PP plates.

For the conventional FSW of PP, it is difficult to find a trend, as shown in Figure 4. In fact, the highest efficiencies of the welds, about 86%, are obtained for *w/v* ratios between 25 rot/mm and 60 rot/mm, see the oval continuous line. On the other hand, the majority of the conventional FSW joints display an efficiency of below 70% and above 30%. A set of results may be observed within an oval dotted line with a joint efficiency of below 30% for *w/v* ratios below 50 rot/mm. It is evident that such a large difference in joint efficiency between these three groups cannot be attributed to the *w/v* ratio, which is similar in the three. Besides, Figure 4 also shows some conventional FSW results, inside a dashed line, with a joint efficiency of below 40%, although the *w/v* ratio is equal to or greater than 100. The high dispersion observed in Figure 4 suggests that in conventional FSW, in addition to the tool rotation and welding speeds, other factors have a decisive influence on the efficiency of the welds. The analysis of other factors, such as the tool tilt angle or the tool geometry, made it possible to highlight the influence of the tool pin geometry in the case of conventional FSWs. The effect of the tool pin geometry on the joint efficiency achieved by welds performed with different *w/v* ratios is shown in Figure 5.

PP plates of 10 mm thick were welded by Lenin et al. [60], using tools with square or triangular prismatic pins, conical (also called taper) pins and cylindrical pins with or without thread for different *w/v* ratios. The welds made with the cylindrical threaded pin tool achieved the 4 highest joint efficiencies in Figure 5, between 79% and 86%. The optimum tool rotational and welding speeds determined were 1500 rpm and 60 mm/min, respectively, which corresponds to a *w/v* ratio of only 25 rot/mm. The authors also claimed that the pin geometry has an influence of about 50% on the final weld quality. With the tool of square pin, a maximum strength of about 75% of the base material strength was obtained for *w/v* = 40 rot/mm, as illustrated in Figure 5.

Conventional FSW using square prismatic, cylindrical and conical pin geometries, all unthreaded, was performed by Sahu et al. [61] to butt weld 6 mm thick PP plates. According to the authors, the results achieved with the conical pin showed that the material was not properly mixed, and, consequently, a void was formed in the stir zone. These welds could be broken easily by hand and therefore, the author decided to omit the values of the joint efficiency for this pin geometry. In this study, the highest joint efficiency of about 60% was obtained with the prismatic square pin geometry, a rotational speed of 750 rpm and a welding speed of 15 mm/min, which corresponds to 50 rot/mm, because a good mixing of the material was guaranteed without overheating the polymer. The same welding and rotational speeds led to the highest joint efficiency achieved for the cylindrical pin of 56%. In general, the square pin performed welds with better tensile strength than the cylindrical pin, because it provides a better mixture of the molten material. For tools with square or cylindrical pin and very high tool rotation speeds (see dashed line of Figure 5) a reduction in weld efficiency is observed, due to the excess of heat generated.

The diameter of the tool shoulder also has a significant influence on the quality of the welds performed with conventional FSW tool. Increasing the diameter of the tool shoulder increases the heat generated in the process, which increases the distortion of the welded parts, as well as the material peeling rate of the weld surface, as mentioned by Sahu et al. [61].

Comparing Figure 3 and Figure 4, there is an obvious difference in the trend for variation in joint efficiency with the *w/v* ratio between PE welds and PP welds. In fact, the joint efficiency increases with the *w/v* ratios of up to about 120 in most PE welds, in contrast to the PP welds, where the highest joint efficiencies are obtained for *w/v* ratios below 60 rot/mm. Given that the welding technologies used are identical, this behavior may result from differences in the structure and/or in the thermal properties of these polymer families, because the mechanical strength and ductility of the welds depend on the heat input in the process [26]. The mechanical strength increases with the degree of crystallinity of the weld [62], which is influenced by the temperature. PP has lower thermal conductivity and specific heat than PE, so it should require a lower *w/v* ratio to provide adequate heat input to the polymer during welding. The presence of the weld root defect is influenced by the low thermal conductivity of the polymers and reduces the efficiency of the joint. This aspect is, however, little analyzed in most articles.

### 3.2. Double-Side FSW

The effect of double-side passing the tool was studied by Arici and Sinmaz [47]. These researchers verified that conventional FSW, i.e., with a single passage of the tool on 3 mm thick medium density polyethylene (MDPE) plates, in butt joint configuration, led to the formation of the root defect. The thickness of these defects was 0.2 mm, which was equal to the difference between the thickness of the plate and the length of the cylindrical pin (2.8 mm). Because of this, the joints failed easily and could even be broken by hand. To evaluate the potential of a double-side passage of the tool, one in each side of the plate, to remove this defect, these authors double-side welded 5 mm thick MDPE plates with the same tool used before, with a pin length of 2.8 mm and 5 mm in diameter. Later, Arici and Selale [63] also performed double-side FSW on 5 mm thick MDPE plates with this same tool. In both cases, all the welds performed with a double passage did not present root defects and achieved better results in tensile and flexural strength tests. The maximum joint efficiency of the new technique was about 87% of the tensile strength of the base material, for a *w/v* ratio of 80 rot/mm, see Figure 3.

The double-side passage of the tool was also studied by Saeedy and Givi [39], who welded 8 mm thick HDPE plates in butt joint configuration with single and double-side FSW and achieved similar results. Tensile strength and elongation were always higher than those obtained with the single side method, once again, due to the removal of the root defect. While the single side passage achieved a maximum joint efficiency of 69%, the double-side passage of the tool achieved 81% for a *w/v* ratio of about 133 rot/mm, see Figure 3. They also observed that the crystallinity of the joint increased in the double-side welded samples, which resulted in higher impact strength. According to the authors, although the double-pass technique takes twice the time of welding on just one side, the costs of the two techniques are approximately the same. Although the authors do not mention any reason for this, the fact that the process is more efficient should lead to a lower number of welds being rejected, compensating for the increase in welding time. Figure 3 shows that for welds with a double-side pass the joint efficiency grows with the *w/v* ratio but even for low *w/v* ratio the joint efficiency is greater than 55%, which shows that the procedure is effective to remove the root defect. This proves the importance that the removal of the root defect has in the improvement of the weld strength.

### 3.3. FSW with Stationary Shoulder Tool

For the comparison of the results of conventional FSW and those related to stationary shoulder FSW for PE and PP, see Figure 3 and Figure 4, which show that most of the stationary shoulder joint efficiencies are above the conventional ones. This means that in general terms, the absence of rotation from the shoulder leads to higher joint efficiency. This behaviour is related to the better surface quality and/or the absence of internal defects in the welds produced with a stationary shoulder. These aspects depend on other factors too, such as the material to be welded or other welding parameters, as shown below.

FSW using a stationary shoulder tool was studied by Eslami et al. [26] to butt weld 3 mm thick HMW-PE plates. The shoulder was made of Teflon and a copper sleeve was used around the pin. Most of their experiments achieved joint efficiencies above 75% and four of them were above 90%. These 4 points are highlighted in the top left corner of Figure 3, inside the oval continuous line. These results were achieved with *w/v* values between 35 rot/mm and 83 rot/mm. The influence of the axial force, a parameter that is many times forgotten, was also considered in this study. Axial forces of 800 N, 950 N and 1100 N were studied, and the middle value resulted in the highest joint strength. The lowest force was not enough to forge the polymer and the highest force led to an excessive reduction in the thickness of the weld. According to them, the copper sleeve was also important, because it preheats the polymer before the pin action. In fact, although this tool has no external heat input, it generates heat internally, due to the friction between the pin and sleeve. This preheating effect allowed higher welding speeds to be used in the process and contributed to preventing the formation of the root defect due to the increase in the welding temperature. The high joint efficiencies achieved are related with this preheating effect and to the optimisation of the axial force. In this study, two experimental sets, resulting from non-ideal axial force, rotational speed, and welding speed, obtained joint efficiencies of under 50%, as illustrated in Figure 3.

FSW with a stationary shoulder tool was also performed by Moreno-Moreno et al. [59], for butt welding 8.5 mm thick HDPE plates. The heat generation was only the caused by the pin. The non-rotating square wooden shoulder with a support ring-wing made of bronze resulted in smooth surfaces. In this case, a maximum joint efficiency of 91% was achieved with a rotational speed of 1036 rpm and welding speed of 14 mm/min, which means a *w/v* ratio of 74 rot/mm, see Figure 3. This technique produces welds with a good surface finish and free of defects. No voids or cracks were found in the microscopic analysis, which helps to explain the high joint efficiencies achieved. These authors state that the mechanical strength, hardness, and crystallinity of the welds decrease when the speed of rotation of the tool increases, but, in our opinion, not all these conclusions cannot be drawn from the results presented in the article.

A microscopic analysis of the morphology of PP welds produced by FSW was carried out by Kiss and Czigány [44]. Welds were produced with a stationary shoulder tool on 10 mm thick plates using a welding speed of 60 mm/min. The author’s aim was to identify the differences between a weld with a high tensile strength and another with a low one. The strongest weld, with a joint efficiency of 86%, was produced with a rotational speed of 3000 rpm (*w/v* = 50 rot/mm) and the weakest, with a joint efficiency of 54.9%, was achieved with 2000 rpm (*w/v* = 33.3 rot/mm). These two welds are represented in Figure 4. Therefore, from this figure it should be concluded that the stationary shoulder allowed welds with high joint efficiency, but small difference in *w/v* ratio can ruin the results. The authors mention that from the microscopic point of view, the same spherulitic structure was found in the base material and in the center of the weld seam, but the spherulites of the base material are larger than those obtained in center of the seam. The size of the spherulites of the two welds was approximately the same, which may be a consequence of similar cooling conditions. The existence of a transition zone between the base material and the weld seam is also observed. The thickness of the transition zone was bigger for the weakest joint. The authors also conclude that smaller weld seams with less complex morphologies result in better joint efficiencies of the FSW process.

### 3.4. FSW of Preheated Polymer

By preheating the polymers, the amount of heat generated by friction required for the welding process is reduced. This technique may not be suitable for all applications, because the process of heating the polymer is slower than for metals. Even so, the results illustrated in Figure 3 show that this technique makes it possible to achieve higher joint efficiencies for lower *w/v* ratio than with conventional FSW. This was understandable because if the heat required, generated by friction, is reduced, the rotational speed may be decreased, or the welding speed increased, to achieve the same heating temperature as the conventional process.

For the FSW of 4 mm thick ultra-high molecular density polyethylene (UHMW-PE) plates in butt joint configuration, Aydin [3] tried to increase the joint efficiency by increasing the rotational speed of the tool, but achieved a maximum efficiency of about 72%. He observed that this procedure led to the formation of large defects, rough surfaces, and the burning of the polymer. Further, by increasing the welding speed, the expulsion of material also increased. These attempts led to a decrease in the strength of the joint. After observing these difficulties, he tested another solution, which was the effect of preheating the plates. As a result, the voids and cavities formed during the conventional FSW were removed and defect-free joints with smoother surfaces were achieved. The joint efficiency of these welds increased up to 89% with a preheating temperature of 50 °C, a tool rotational speed of 960 rpm and a welding speed of 20 mm/min, which corresponds to a *w/v* ratio of 48 rot/mm, see Figure 3. Therefore, these results proved that preheating the polymer improved the quality and strength of the welds. The welds done using the same welding parameters but preheating the polymer to a temperature of 80 °C, presented a joint efficiency of around 72%, proving that excessive heating is harmful.

### 3.5. FSW with Conventional Heated Tool

Preheating large plates of polymer is difficult to do and maintain during the welding so, the supply of heat through a heated tool is a more practical and quicker solution. The heating of the tool is usually done by induction or by applying electrical resistances inside the tool. This technology gives different results in terms of strength of welds in PE and PP, but, as the results are scarce, they are presented together.

A new FSW hot tool technique named induction friction stir welding (i-FSW) was developed and presented by Vijendra and Sharma [64]. They used an induction coil around the tool to heat it and a temperature sensor that measures and controls the temperature of the tool during the welding process. This new technique was used to butt weld HDPE plates of 5 mm thick. They observed that when no external heat was supplied the maximum joint efficiency achieved was about 50% for a rotational speed of 3000 rpm and a welding speed of 50 mm/min, which means a *w/v* ratio of 60 rot/mm. By heating the tool up to 450 and 550 °C for 2000 rpm and 50 mm/min, the joint efficiency was 104.3% and 98.7% respectively, see Figure 3. It is concluded, therefore, that the new tool design and the supply of heat bring improvements in the quality and strength of the welds performed by i-FSW. Despite the maximum joint efficiency reached, about 100%, the experiments were conducted in bead-on-plate (BOP) configuration, which reduces the likelihood of root defects forming.

Another new assisted heating tool with a rotary shoulder, that had a 50 W electrical resistance inside, was developed and presented by Banjare et al. [65]. This tool was created in order to avoid major changes in the conventional tool design. This new tool allowed improvements in tensile and impact strengths in butt welds of 5 mm thick PP plates. The results demonstrated that the use of this tool heated at 110 °C brings advantages to the process, since the tensile strength and elongation of the welds were always superior to those obtained by the non-heated tool at the same rotational speed. The welds obtained without a heated tool showed large propensity to form cavities within the seam and irregular surfaces, with pronounced burr defects, while the welds obtained with the heated tool presented few or no defects and a better surface finish. The maximum tensile strength was obtained for 720 rpm, while the highest elongation was observed for 360 rpm. Due to the similarity of the results obtained in terms of strength, both rotational speeds are suggested for future studies. For the *w/v* ratio in the range 18–27 rot/mm, the maximum joint efficiencies for the FSWs with and without the heated tool were 56% and 29%, respectively. The values of the experiments done without heating the tool are inside the dotted line in Figure 4. Although these results are poor, if they are compared with those of conventional FSW from other studies, they demonstrate the benefits of using an external heat source once again.

### 3.6. FSW with Tool of Heated Stationary Shoulder

A hot tool with a stationary shoulder is a combination of two FSW techniques and it was the welding technique that allowed the highest values of joint efficiency for both materials. This is because this technique joins the benefits of stationary shoulders and the application of external heat. The most common tool used in this technique is sometimes named hot shoe. The hot shoe tool was a solution found to solve the problems related to the formation of voids within the weld seam and also to improve the mixing of the molten material in the FSW of polymeric materials [66]. This tool was developed and patented by Nelson et al. [48] at Brigham Young University. The hot shoe consists of a long rectangular stationary shoulder, a rotating pin and a heating system inserted inside the shoulder [58]. According to Strand [15], it is important to guarantee a uniform cooling rate in order to avoid the formation of defects during the solidification phase. If the outer material cools faster than the inner material, a hard shell with a still softened core is formed. During the solidification, the inner layers shrink creating voids. With the implementation of the hot shoe in the process, the cooling and solidification occurred under pressure in a longer period. Thus, the natural shrinkage of the material is reduced and, therefore, there is less probability to form voids.

#### 3.6.1. Welds in Polyethylene

Joining of 10 mm thick HDPE plates by FSW, using a hot shoe tool coated with PTFE was studied by Azarsa et al. [58] and Mostafapour and Azarsa [38]. In this study, the friction was still the main source of the heat generated and the hot shoulder was mainly responsible for slowing the cooling stage. The stationary shoe was used to prevent the materials from being expelled better than conventional rotating shoulders do. In this experiment, different rotational speeds and welding speeds were used and different shoe temperatures were also tested. The results showed that the increase in the shoe temperature from 80 °C to 140 °C led to an increase in the tensile strength of the joints. Microstructural observation also showed that the polymer degrades as a result of the stirring action of the pin at 80 °C. By increasing the tool’s temperature, this defect was removed. Joint efficiencies above 90% were achieved with tool temperatures of 110 °C and 140 °C, and rotational speeds of 1600 rpm and 1250 rpm and welding speed of 25 mm/min. This means *w/v* ratios of 64 rot/mm and 50 rot/mm. These researchers also tested the effect of not coating the shoe with PTFE and observed that the coating is important concerning the quality and strength of the weld. Without the coating, the material stuck to the shoe and a bad surface finish was formed. As a result, residual stress concentration was formed on the top of the weld. By coating the shoulder surface, these defects were removed, and very smooth and regular surfaces were obtained. Figure 3 shows that the tool with heated stationary shoulder performs welds with joint efficiencies higher than the majority of those obtained with the other welding processes mentioned above.

In another study, using the same material (10 mm thick HDPE) and welding process (hot shoe), Azarsa and Mostafapour [67] investigated the effect of heating temperatures between 70 °C and 150 °C for several tool rotational and welding speeds on the weld’s quality. They observed that at rotational speeds above 1400 rpm the polymer began to burn in the preliminary tests. Below 700 rpm, the heat generated was insufficient to ensure adequate material mixing, and wormhole defects were formed. At welding speeds above 100 mm/min, the quality of the weld was bad, and the weld crown became filled with deformations and external voids. The output results do not contain information about the tensile strength of the welds, and, because of this, it was not possible to compare the results from these experiments with other studies directly. Even so, the results showed that a maximum flexural strength of 96% was achieved with a tool temperature of 110 °C, a rotational speed of 1400 rpm and a welding speed of 25 mm/min. This means a *w/v* ratio of 56 rot/mm, which is in accordance with the optimal values determined for this parameter in the abovementioned study.

#### 3.6.2. Welds in Polypropylene

A new heated tool with a stationary shoulder was presented and tested by Moochani et al. [46] for butt welding 4 mm thick PP plates. This tool was heated with a hot air gun while the temperature was measured with an infrared sensor. According to this researcher, the advantage of using this tool is that the temperature can be maintained without changing other welding parameters. In the preliminary testing phase, the researchers compared the FSW process with and without heating the tool, using a rotational speed of 950 rpm and a welding speed of 24 mm/min (*w/v* ≈ 39.6 rot/mm). The welding with a heated tool resulted in joints with a better surface finish and higher mechanical strength. The effectiveness of this tool was proven for the welding of PP, since a joint efficiency of 96% and an elongation of 98% of the base material were obtained in this study. This was explained by the improvement obtained in the polymeric mixture during the process. The tensile tests also revealed that an increase in the temperature of the tool from 130 °C to 150 °C resulted in an improvement in the tensile strength and an increase in the maximum elongation. In turn, an increase in the temperature of the tool to 170 °C led to a slight reduction in the tensile strength and to a big drop in the maximum elongation, caused by an excessive reduction in the crystallinity of the weld. Tensile strengths and elongations above 90% were achieved with different welding conditions. A maximum joint efficiency of 96% was achieved with a rotational speed of 565 rpm, a welding speed of 24 mm/min, which means *w/v* ratio of about 23.5 rot/mm, and a tool temperature of 150 °C. On the other hand, by using the Taguchi method and the analysis of variance, the best parameters found were a rotational speed of 950 rpm, a welding speed of 24 mm/min, which means *w/v* ratio of about 39.6 rot/mm, and a tool temperature of 150 °C. However, no confirmatory tests have been carried out to support these claims. Figure 4 shows that this welding process produces more resistant welds than any of the other techniques mentioned, as it was for the PE.

### 3.7. FSW in Other Polymeric Materials

The conclusions presented in the current work for PE and PP are similar to those drawn for other materials such as ABS or PA6, although the number of studies available in the literature is much smaller. Mendes et al. [30] butt welded 6 mm thick ABS plates with a stationary shoe, with and without heating. The study performed with and without a heated tool achieved a joint efficiency of 75.5% and 68%, respectively. These researchers also studied the influence of the axial force and found that a minimum value of pressure is required to ensure defect-free welds. Bagheri et al. [29] reported a maximum joint efficiency of 88.8% with a hot shoe while welding 5 mm tick ABS sheets. Zafar et al. [68] and Husain et al. [34] butt welded 16 mm and 8 mm thick plates of PA6 and achieved joint efficiencies of 32% and 55% with conventional FSW, respectively. On the other hand, Mostafapour and Asad [35] butt welded 6 mm thick plates of the same material with a hot shoe tool and achieved a joint efficiency of 98%. These results reinforce the conclusions obtained for PE and PP that hot tools with stationary shoulders are better to weld polymeric materials and that the influence of parameters often neglected, such as axial force, should not be underestimated.

Recently Derazkola et al. [69] proved experimentally in PC welding that the use of underwater friction-stir welding (UFSW) reduces the heat input in the process, which decreases structural changes in the polymer and the formation of defects and increases the tensile strength of the welds. Furthermore, they did the thermo-mechanical modelling of the process. Other new technologies have been developed recently, based on FSW, but mainly for dissimilar joining, as is the case of fed friction stir processing (FFSP) in AA6062/PMMA bindings [70]. The authors argue that with the introduction of alumina nanoparticles during FFSP the tensile strength of these bonds can be increased up to 14%.

## 4. Discussion, Conclusions, and Future Work

From the literature, it is clear that the application of the FSW process to polymers, specifically polyethylene and polypropylene, presents a lot of potential, but it still needs to be more investigated in the future. FSW with conventional tools makes welds with a satisfactory tensile strength, even if most of the authors reported a maximum joint efficiency of under 75%. Joint efficiency increases with the tool rotational to traverse speeds ratio (*w/v*) for the conventional process, although it is also affected by other process parameters. A high *w/v* ratio does not in itself guarantee that high strength welds are obtained. The rotational speed of the tool must be above a certain limit, but excessive rotational speeds can degrade the polymer. In addition, the rotation of the tool shoulder causes degradation of the polymer surface, which seems difficult to overcome using these tools.

The use of stationary shoulder tools allowed better weld surface finish, fewer defects, and higher joint efficiency. Figure 3 shows joint efficiencies above 90% for stationary shoulder tools, but, in this case, it should also be considered the heat generated in the tool due to the friction between the copper sleeve and the rotating probe [26]. The use of external heat sources makes welds with a greater tensile strength possible, as a result of the improvement in the material mixing and the removal of root defects and cavities in the retreating side of the welds. The technique of preheating the polymers to be welded improves the resistance of the welds, but it is impractical, especially for long welds. Conventional heated tools also provide a significant improvement in joint efficiency, but the results available are scarce and, in our opinion, the detrimental effect of shoulder rotation is difficult to overcome.

The use of heated stationary shoulders allows the production of welds with the highest joint efficiency because they join the benefits of the application of external heat and of non-rotating shoulder. The current authors doubt that the system is effective for high polymer thicknesses, due to the reduced thermal conductivity of the polymers. In this case, heating the probe can be an interesting option, as it must be more effective in heating the entire thickness of the polymer. The double-side passage should be used when it is intended to guarantee the removal of the root defect. Table 1 summarizes the main characteristics, potential and limitations of each welding technique. Its analysis reinforces the idea that the FSW techniques with Heated Stationary Shoulder tools provide welds with better quality and joint efficiency. However, as mentioned above, due to the low thermal conductivity of polymers, the heat input to the pin (and not just to the shoulder) must be considered in the future, especially for welding thicker plates.

Figure 3 and Figure 4 show that the mechanical behaviour of welds in PE and PP is markedly different for any of the FSW technologies and parameters used, which suggests that this is due to the different physical and mechanical properties of the polymers. Zafar et al. [71] showed, based on the literature, that the optimum joining parameters are very different from one polymer to another, and attributed this behavior to differences in the melt viscosity of the polymers. These aspects need to be further investigated. This study also shows that, in the FSW of polymers, there are several features that are still poorly studied, requiring further analysis. Some topics that deserve reference are the following:

- The quantification of the heat generated in the welding of polymers and the contribution of the heat added externally are still undefined.
- The effect of the plunge depth or axial force on the morphology and strength of the welds was not discussed in most of the works and needs further research.
- There are still only a few studies that analyse the microstructure and degree of crystallinity of the welds and their relationship with the welding parameters, so this aspect must be studied further.
- The relationship between the physical properties of the polymers and the optimal welding parameters needs careful analysis.
- Heated Stationary Shoulder tools are poorly adapted for welding on three-dimensional paths, so it is necessary to rethink the design of the tools for these applications.

## Figures and Tables

**Table 1:** Main characteristics, potential, and limitations of each Friction Stir Welding Technique.

Friction Stir Welding Technique	Joint Efficiency	Surface Finish	Material Mixing Quality	Root Defect Formation	Internal Defects Formation	Implementation
Conventional Tool	Satisfactory	Poor	Normal	Likely	Almost Always	Very Easy
Double-side Pass (Conventional tool)	Good	Poor	Normal	unlikely	Almost Always	Easy
Preheated Polymer	Good	Fair	Good	Reduced	Less likely	Difficult
Conventional Heated Tool	Good	Fair	Good	Reduced	Less likely	Easy
Stationary Shoulder	Very Good	Very Good	Good	Reduced	Less likely	Easy
Heated Stationary Shoulder	Excellent	Excellent	Very Good	Reduced	Less likely	Easy
i-FSW	Very Good	Fair	Good	Reduced	Less likely	Easy

## Data Availability

Data derived from public domain resources.

## References

[1] Strand S. Joining plastics—Can Friction Stir Welding compete?. Proceedings of the Electrical Insulation Conference and Electrical Manufacturing and Coil Winding Conference and Exhibition.

[2] Mishra D., Sahu S.K., Mahto R.P., Pal S.K., Pal K., Dixit U., Narayanan R. (2019). Friction Stir Welding for Joining of Polymers. Strengthening and Joining by Plastic Deformation.

[3] Aydin M. (2010). Effects of welding parameters and pre-heating on the friction stir welding of UHMW-polyethylene. Polym. Plast. Technol. Eng..

[4] Amancio-Filho S.T., Dos Santos J.F. (2009). Joining of polymers and polymer-metal hybrid structures: Recent developments and trends. Polym. Eng. Sci..

[5] Awaja F., Gilbert M., Kelly G., Fox B., Pigram P.J. (2009). Adhesion of polymers. Prog. Polym. Sci..

[6] Amanat N., James N.L., McKenzie D.R. (2010). Welding methods for joining thermoplastic polymers for the hermetic enclosure of medical devices. Med. Eng. Phys..

[7] Fernandes F.A.O., Pereira A.B., Guimarães B., Almeida T. (2020). Laser welding of transmitting high-performance engineering thermoplastics. Polymers.

[8] Vazquez-Martinez J.M., Piñero D., Salguero J., Batista M. (2020). Evaluation of the joining response of biodegradable polylactic acid (PLA) from fused deposition modeling by infrared laser irradiation. Polymers.

[9] Qiu J., Zhang G., Sakai E., Liu W., Zang L. (2020). Thermal welding by the third phase between polymers: A review for ultrasonicweld technology developments. Polymers.

[10] Ahmed T.J., Stavrov D., Bersee H.E.N., Beukers A. (2006). Induction welding of thermoplastic composites-an overview. Compos. Part A Appl. Sci. Manuf..

[11] Lambiase F., Paoletti A., Di Ilio A. (2017). Friction spot stir welding of polymers: Control of plunging force. Int. J. Adv. Manuf. Technol..

[12] Pereira A.B., Fernandes F.A.O., de Morais A.B., Quintão J. (2019). Mechanical strength of thermoplastic polyamide welded by Nd:YAG laser. Polymers.

[13] Oliveira P.H.F., Amancio-Filho S.T., Dos Santos J.F., Hage E. (2010). Preliminary study on the feasibility of friction spot welding in PMMA. Mater. Lett..

[14] Zhu X., Liu G., Guo Y., Tian Y. (2007). Study of PMMA thermal bonding. Microsyst. Technol..

[15] Strand S. (2004). Effects of Friction Stir Welding on Polymer Microstructure. Master’s Thesis.

[16] Thomas W.M., Nicholas E.D., Needham J.C., Murch M.G., Templesmith P., Dawes C.J. (1991). Friction Stir Butt Welding. International Patent.

[17] Thomas W., Nicholas E. (1997). Friction stir welding for the transportation industries. Mater. Des..

[18] Payganeh G.H., Mostafa Arab N.B., Dadgar Asl Y., Ghasemi F.A., Saeidi Boroujeni M. (2011). Effects of friction stir welding process parameters on appearance and strength of polypropylene composite welds. Int. J. Phys. Sci..

[19] Squeo E.A., Bruno G., Guglielmotti A., Quadrini F. (2009). Friction stir welding of polyethylene sheets. Ann. DUNĂREA JOS Univ. Galati Fascicle V Technol. Mach. Build..

[20] Mishra R.S., Ma Z.Y. (2005). Friction stir welding and processing. Mater. Sci. Eng. R Rep..

[21] Huang Y., Meng X., Wang Y., Xie Y., Zhou L. (2018). Joining of aluminum alloy and polymer via friction stir lap welding. J. Mater. Process. Technol..

[22] Thomas W.M., Johnson K.I., Wiesner C.S. (2003). Friction Stir Welding—Recent Developments in Tool and Process Technologies. Adv. Eng. Mater..

[23] Rezgui M.A., Ayadi M., Cherouat A., Hamrouni K., Zghal A., Bejaoui S. (2010). Application of Taguchi approach to optimize friction stir welding parameters of polyethylene. EPJWC.

[24] Inaniwa S., Kurabe Y., Miyashita Y., Hori H. (2013). Application of friction stir welding for several plastic materials. Proceedings of the 1st International Joint Symposium on Joining and Welding.

[25] Bozkurt Y. (2012). The optimization of friction stir welding process parameters to achieve maximum tensile strength in polyethylene sheets. Mater. Des..

[26] Eslami S., Miranda J.F., Mourão L., Tavares P.J., Moreira P.M.G.P. (2018). Polyethylene friction stir welding parameter optimization and temperature characterization. Int. J. Adv. Manuf. Technol..

[27] Hussain R.K., Majeed A.A. (2018). Thermoplastics Polymers Friction Stir Welding: Review. Int. J. Eng. Technol..

[28] Stokes V.K. (1989). Joining methods for plastics and plastic composites: An overview. Polym. Eng. Sci..

[29] Bagheri A., Azdast T., Doniavi A. (2013). An experimental study on mechanical properties of friction stir welded ABS sheets. Mater. Des..

[30] Mendes N., Loureiro A., Martins C., Neto P., Pires J.N. (2014). Morphology and strength of acrylonitrile butadiene styrene welds performed by robotic friction stir welding. Mater. Des..

[31] Mendes N., Loureiro A., Martins C., Neto P., Pires J.N. (2014). Effect of friction stir welding parameters on morphology and strength of acrylonitrile butadiene styrene plate welds. Mater. Des..

[32] Pirizadeh M., Azdast T., Rash Ahmadi S., Mamaghani Shishavan S., Bagheri A. (2014). Friction stir welding of thermoplastics using a newly designed tool. Mater. Des..

[33] Panneerselvam K., Lenin K. (2014). Joining of Nylon 6 plate by friction stir welding process using threaded pin profile. Mater. Des..

[34] Husain I.M., Salim R.K., Azdast T., Hasanifard S., Shishavan S.M., Lee R.E. (2015). Mechanical properties of friction-stir-welded polyamide sheets. Int. J. Mech. Mater. Eng..

[35] Mostafapour A., Taghizad Asad F. (2016). Investigations on joining of Nylon 6 plates via novel method of heat assisted friction stir welding to find the optimum process parameters. Sci. Technol. Weld. Join..

[36] Aghajani Derazkola H., Simchi A., Lambiase F. (2019). Friction stir welding of polycarbonate lap joints: Relationship between processing parameters and mechanical properties. Polym. Test..

[37] Lambiase F., Grossi V., Paoletti A. (2020). Effect of tilt angle in FSW of polycarbonate sheets in butt configuration. Int. J. Adv. Manuf. Technol..

[38] Mostafapour A., Azarsa E. (2012). A study on the role of processing parameters in joining polyethylene sheets via heat assisted friction stir welding: Investigating microstructure, tensile and flexural properties. Int. J. Phys. Sci..

[39] Saeedy S., Givi M.K.B. (2010). Experimental investigation of double side friction stir welding (FSW) on high density polyethylene blanks. American Society of Mechanical Engineers Digital Collection, Proceedings of the ASME 2010 10th Biennial Conference on Engineering Systems Design and Analysis, ESDA2010, Istanbul, Turkey, 12–14 July 2010.

[40] Sharma A.K.R., Roy Choudhury M., Debnath K. (2020). Experimental investigation of friction stir welding of PLA. Weld. World.

[41] Aghajani Derazkola H., Simchi A. (2018). Experimental and thermomechanical analysis of the effect of tool pin profile on the friction stir welding of poly(methyl methacrylate) sheets. J. Manuf. Process..

[42] Elyasi M., Derazkola H.A. (2018). Experimental and thermomechanical study on FSW of PMMA polymer T-joint. Int. J. Adv. Manuf. Technol..

[43] Adibeig M.R., Hassanifard S., Vakili-Tahami F., Hattel J.H. (2018). Experimental investigation of tensile strength of friction stir welded butt joints on PMMA. Mater. Today Commun..

[44] Kiss Z., Czigány T. (2012). Microscopic analysis of the morphology of seams in friction stir welded polypropylene. Express Polym. Lett..

[45] Sharma R., Singh O.P. (2013). Effect of FSW Process Parameters on Mechanical Properties of Polypropylene: An Experimental Study. Int. J. Innov. Res. Sci. Eng. Technol..

[46] Moochani A., Omidvar H., Ghaffarian S.R., Goushegir S.M. (2019). Friction stir welding of thermoplastics with a new heat-assisted tool design: Mechanical properties and microstructure. Weld. World.

[47] Arici A., Sinmaz T. (2005). Effects of double passes of the tool on friction stir welding of polyethylene. Proceedings of the Journal of Materials Science.

[48] Nelson T.W., Sorenson C.D., Johns C.J. (2004). Friction Stir Welding of Polymeric Materials. U.S. Patent.

[49] Mendes N., Neto P., Simão M.A., Loureiro A., Pires J.N. (2016). A novel friction stir welding robotic platform: Welding polymeric materials. Int. J. Adv. Manuf. Technol..

[50] Lambiase F., Derazkola H.A., Simchi A. (2020). Friction stir welding and friction spot stir welding processes of polymers-state of the art. Materials.

[51] Huang Y., Meng X., Xie Y., Wan L., Lv Z., Cao J., Feng J. (2018). Friction stir welding/processing of polymers and polymer matrix composites. Compos. Part A Appl. Sci. Manuf..

[52] Eslami S., Tavares P.J., Moreira P.M.G.P. (2017). Friction stir welding tooling for polymers: Review and prospects. Int. J. Adv. Manuf. Technol..

[53] Mendes N., Neto P., Loureiro A., Moreira A.P. (2016). Machines and control systems for friction stir welding: A review. Mater. Des..

[54] Iftikhar S.H., Mourad A.-H.I., Sheikh-Ahmad J., Almaskari F., Vincent S. (2021). A Comprehensive Review on Optimal Welding Conditions for Friction Stir Welding of Thermoplastic Polymers and Their Composites. Polymers.

[55] Saeedy S., Givi M.K.B. (2011). Investigation of the effects of critical process parameters of friction stir welding of polyethylene. Proc. Inst. Mech. Eng. Part B J. Eng. Manuf..

[56] Saeedy S., Givi M.K.B. Experimental application of friction stir welding (FSW) on thermo plastic medium density polyethylene blanks. Proceedings of the ASME 2010 10th Biennial Conference on Engineering Systems Design and Analysis, ESDA2010, ASMEDC.

[57] Nateghi E., Hosseinzadeh M. (2016). Experimental investigation into effect of cooling of traversed weld nugget on quality of high-density polyethylene joints. Int. J. Adv. Manuf. Technol..

[58] Azarsa E., Asl A.M., Tavakolkhah V. (2012). Effect of process parameters and tool coating on mechanical properties and microstructure of heat assisted friction stir welded polyethylene sheets. Adv. Mater. Res..

[59] Moreno-Moreno M., Macea Romero Y., Rodríguez Zambrano H., Restrepo-Zapata N.C., Afonso C.R.M., Unfried-Silgado J. (2018). Mechanical and thermal properties of friction-stir welded joints of high density polyethylene using a non-rotational shoulder tool. Int. J. Adv. Manuf. Technol..

[60] Lenin K., Shabeer H.A., Suresh Kumar K., Panneerselvam K. (2014). Process Parameters Optimization for Friction Stir Welding of Polypropylene Material Using Taguchi’s Approach.

[61] Sahu S.K., Mishra D., Mahto R.P., Sharma V.M., Pal S.K., Pal K., Banerjee S., Dash P. (2018). Friction stir welding of polypropylene sheet. Eng. Sci. Technol. Int. J..

[62] Humbert S., Lame O., Vigier G. (2009). Polyethylene yielding behaviour: What is behind the correlation between yield stress and crystallinity?. Polymer.

[63] Arici A., Selale S. (2007). Effects of tool tilt angle on tensile strength and fracture locations of friction stir welding of polyethylene. Sci. Technol. Weld. Join..

[64] Vijendra B., Sharma A. (2015). Induction heated tool assisted friction-stir welding (i-FSW): A novel hybrid process for joining of thermoplastics. J. Manuf. Process..

[65] Banjare P.N., Sahlot P., Arora A. (2017). An assisted heating tool design for FSW of thermoplastics. J. Mater. Process. Technol..

[66] Dashatan S.H., Azdast T., Ahmadi S.R., Bagheri A. (2013). Friction stir spot welding of dissimilar polymethyl methacrylate and acrylonitrile butadiene styrene sheets. Mater. Des..

[67] Azarsa E., Mostafapour A. (2014). Experimental investigation on flexural behavior of friction stir welded high density polyethylene sheets. J. Manuf. Process..

[68] Zafar A., Awang M., Khan S.R., Emamian S. (2015). Effect of Double Shoulder Tool Rotational Speed on Thermo-Physical Characteristics of Friction Stir Welded 16mm Thick Nylon6. Appl. Mech. Mater..

[69] Aghajani Derazkola H., Garcia E., Elyasi M. (2021). Underwater friction stir welding of PC: Experimental study and thermo-mechanical modelling. J. Manuf. Process..

[70] Aghajani Derazkola H., Simchi A. (2020). A new procedure for the fabrication of dissimilar joints through injection of colloidal nanoparticles during friction stir processing: Proof concept for AA6062/PMMA joints. J. Manuf. Process..

[71] Zafar A., Awang M., Khan S.R., Awang M. (2017). Friction stir welding of polymers: An overview. Lecture Notes in Mechanical Engineering, Proceedings of the 2nd International Conference on Mechanical, Manufacturing and Process Plant Engineering, Kuala Lumpur, Malaysia, 23–24 November 2016.

